# Breast cancer risk in a screening cohort of Asian and white British/Irish women from Manchester UK

**DOI:** 10.1186/s12889-018-5090-9

**Published:** 2018-01-25

**Authors:** D. Gareth Evans, Adam R. Brentnall, Michelle Harvie, Susan Astley, Elaine F. Harkness, Paula Stavrinos, Louise S. Donnelly, Sarah Sampson, Faiza Idries, Donna Watterson, Jack Cuzick, Mary Wilson, Anil Jain, Fiona Harrison, Anthony J. Maxwell, Anthony Howell

**Affiliations:** 1 Department of Genomic Medicine, Division of Evolution and Genomic Science, MAHSC, University of Manchester, Central Manchester NHS Foundation Trust, Oxford Road, Manchester, UK; 2 Division of Informatics, Imaging and Data Sciences, Faculty of Biology, Medicine and Health, University of Manchester, Manchester Academic Health Science Centre, Oxford Road, Manchester, UK; 3 Nightingale & Prevention Breast Cancer Centre, University Hospital of South Manchester, Manchester, UK; 4 Centre for Cancer Prevention, Wolfson Institute of Preventive Medicine, Queen Mary University of London, London, UK; 5 School of Medical Sciences, University of Manchester, Oxford Road, Manchester, UK; 6 The Christie, Manchester, UK; 7 Department of Genomic Medicine, MAHSC, St Mary’s Hospital, Central Manchester NHS Foundation Trust, Manchester, M13 9WL UK

**Keywords:** Breast cancer, Risk factors, Ethnicity, United Kingdom

## Abstract

**Background:**

The differences between breast cancer risk factors in white British/Irish and Asian women attending screening in the UK are not well documented.

**Methods:**

Between 2009-15 ethnicity and traditional breast cancer risk factors were self-identified by a screening cohort from Greater Manchester, with follow up to 2016. Risk factors and incidence rates were compared using age-standardised statistics (European standard population).

**Results:**

Eight hundred and seventy-nine Asian women and 51,779 unaffected white British/Irish women aged 46-73 years were recruited. Asian women were at lower predicted breast cancer risk from hormonal and reproductive risk factors than white British/Irish women (mean 10 year risk 2.6% vs 3.1%, difference 0.4%, 95%CI 0.3-0.5%). White British/Irish women were more likely to have had a younger age at menarche, be overweight or obese, taller, used hormone replacement therapy and not to have had children.. However, despite being less overweight Asian women had gained more weight from age 20 years and were less likely to undertake moderate physical activity. Asian women also had a slightly higher mammographic density. Asian age-standardised incidence was 3.2 (95%CI 1.6-5.2, 18 cancers) per thousand women/year vs 4.5 (95%CI 4.2-4.8, 1076 cancers) for white British/Irish women.

**Conclusions:**

Asian women attending screening in Greater Manchester are likely to have a lower risk of breast cancer than white British/Irish women, but they undertake less physical activity and have more adult weight gain.

**Electronic supplementary material:**

The online version of this article (10.1186/s12889-018-5090-9) contains supplementary material, which is available to authorized users.

## Background

The risk of breast cancer, attendant risk factors and the uptake and performance of breast screening are not well understood in ethnic minority groups in the United Kingdom (UK), including those with an Asian ancestry. Screening uptake is likely to be substantially lower in Asian women, and particularly in women of Pakistani or Bangladeshi origin in the UK [1–3]. However, much of the evidence to date has been based on linkage from census data because the National Health Service Breast Screening Programme (NHSBSP) does not collect ethnicity data, nor is it known when Asian women are invited for screening. Breast cancer rates are lower for Asian women in their native countries [4], but appear to become intermediate when Asian women move to higher incidence countries [5]. It is thought that this reflects westernisation of risk factors such as delayed or reduced parity [6]; however, the exact reasons for this remain unclear [4].

The only previous study to have accessed breast cancer risk factors and observed risk in Asian women resident in the UK was in the Million Women Study cohort [16]. This found that recorded risk factors for age at menarche, hormone therapy, alcohol and breast cancer family history, were more protective amongst Asian women compared with white women, whilst Body Mass Index (BMI) was comparable. Additional adjustment for these risk factors for the disease showed that breast cancer incidence was similar to that of white women; these risk factors accounted for almost all the differences in risk.

The NHSBSP operates a three-yearly screening cycle, inviting all women aged 50-70y, and some women aged 47-49 or 70-73y through an on-going cluster randomized trial. During the 2011-12 screening round of the NHSBSP, overall coverage was 77% [7]. Uptake of routine invitations for women aged 50-70 years was 73% with comparatively lower uptake (68%) in the 71-74 year age group. A total of 15,749 women aged 45 and over had cancers detected by the screening programme in 2011-12, a rate of 8.1 cases per thousand women screened, with the cancer detection rate being highest amongst women over 70 years (13.9 per thousand women screened).

The Greater Manchester Breast Screening Programme (GMBSP) invites women aged 47–73 years in five main areas of Greater Manchester: Tameside, Oldham, Salford, Manchester and Trafford. Within each of these areas there are several local screening sites. Ethnic minorities make up just over 20% of the population of these five areas, about half of which are of Asian ethnicity [8]. Uptake to breast screening in Greater Manchester is typically slightly lower than the national average, being 70% versus 73% of eligible women screened during 2011-12.

The aim of this study was to determine whether there were differences in breast cancer risk factors between screening attendees who identified themselves as “Asian or Asian British” compared with “white British or Irish”, and how this might influence breast cancer rates between the two populations.

## Methods

### Cohort

Recruitment to the Predicting Risk of Cancer at Screening (PROCAS) study was carried out in two phases [9]. In phase one (October 2009–October 2012) all women invited for breast screening in the GMBSP were sent an invitation to participate in the study. As screening is triennial, this meant that all women attending screening during the recruitment period were invited once during this time. In phase two (November 2012-March 2015) women not previously screened were invited; thus women recruited during this phase were substantially younger than women recruited in phase one.

The study was approved by North West 7 Research Ethics Committee – GM Central (reference 09/H1008/81).

### Risk factors

A two-page questionnaire was devised to collect self-reported ethnicity (with categories for Asian or Asian British, black or black British, mixed, white British or Irish, other [with free text]; Jewish Origin or Jewish Ashkenazi) and known breast cancer risk factors which are included in the Tyrer-Cuzick model [9, 10]. This included family history information (number and ages of sisters; current age or age at death of mother; and details of any relatives affected by breast or ovarian cancer), hormonal risk factors (age of menarche and first pregnancy, parity, menopausal status and hormone therapy use) and current weight and height. We also collected information on some additional breast cancer risk factors: weight at age 20 years, from which we determined percentage of adult weight gain (current weight/weight at 20 years) × 100%, alcohol consumption and amount of moderate physical activity in the past week [11].

Women were mailed the questionnaire and a consent form in the interval between their screening invitation and attendance for mammography. Consent for entry to the study was taken at the time of the screening appointment. Questionnaire data was entered onto a study database and using the Tyrer-Cuzick version 6 risk calculator, a 10 year risk score for each individual was automatically produced [10].

Mammographic density was measured at entry by visual assessment for the first 53,000 women enrolled. Two expert readers from a pool of 18 assessed percentage density on each mammographic examination (usually four views: left and right medio-lateral oblique and cranio-caudal) using a 0 to 100% visual analogue scale (VAS), as previously described [12]. The average of all VAS scores for each woman was used in the analysis.

### Breast cancer incidence and vital statuses

Breast cancer diagnoses, tumour characteristics and vital statuses were obtained from the screening programme and a local cancer intelligence service using National Health Service (NHS) numbers. Follow up was censored at either breast cancer diagnosis, death or on 31/12/2016 depending on which occurred earliest. Incidence used invasive cancer and ductal carcinoma in situ diagnoses, which was the pre-defined study endpoint and was done in other analyses of risk factors from this cohort, also partly due to the similarity of risk factors for both types of disease [12].

### Statistical methods

We estimated the percentage uptake to the study amongst Asian women as the relative proportion of Asian women in 5 year age bands (46-49, 50-54 etc) in comparison with the proportion from the 2011 UK Census for the five screening areas of Greater Manchester [8]. This calculation assumed that all women in those age groups would have received both an invitation for mammography and to join the PROCAS study over the study recruitment period.

Age-standardised statistics were obtained by weighting the contribution of each woman to reflect the age distribution in the standard 2013 European population [13]. For risk factors, standardisation was undertaken by year, and by 5 year groups for incidence. Inference for standardised risk factors was based on a non-parametric bootstrap (10,000 resamples), and for standardised incidence a parametric bootstrap (Poisson); empirical 95% confidence intervals were obtained [14, 15]. A linear model was used to predict mammographic density given age and BMI, to investigate if differences between the BMI distributions fully explained density difference between the groups. Age-standardised statistics were obtained for birth cohorts (born < 1950, 1950-9 or ≥1960). Tests for trend between the cohorts used the Cuzick (1985) test for continuous or ordered data, or a logistic regression chi-square test for binary risk factors.

## Results and discussion

During the first phase of recruitment, the majority (70%) of women were aged 50-64 years, with 23% aged 65-73 years and 7% younger than 50 years. Most women were white British/Irish (91.0%), 1.3% were Asian, 4.0% had other ethnicities and 3.7% did not report ethnicity. In the second phase, a larger proportion of younger women were recruited: 43% were aged 46-49 years and 49% aged 50-54 years. The majority were again white British/Irish (89.0%) but there were proportionally more Asian women (3.3%) than in the first phase. Overall, almost the same proportion recruited in each phase stated a preference to be informed of their risk (94.5% vs 95.0%).

In total 57,902 women were recruited to PROCAS; 906 of whom had previously developed breast cancer. A total of 891 (1.54%) women self-identified on their questionnaires as being Asian or Asian British (12 with previous breast cancer), with 52,639 (90.9%) who identified themselves as white British or Irish (830 with previous breast cancer). Proportional uptake of Asian women to PROCAS compared with the assumed Asian women population in Greater Manchester is shown in Table 1.

**Table 1:** Proportion of Asian women in the local invited population compared with those entering PROCAS

Age group (year)	Asian proportion at screening ages in Greater Manchester population (census)	Asian women in PROCAS (%)	Relative proportion entering PROCAS
46-49	8.52%	256 (2.70%)	32%
50-54	7.52%	289 (2.14%)	28%
55-59	6.33%	152 (1.64%)	26%
60-64	6.28%	112 (1.16%)	19%
65-69	6.16%	59 (0.99%)	16%
70-74	4.79%	11 (0.75%)	16%

Overall entry to PROCAS amongst Asian women was lower than would be expected in the invited population as a whole. The relative uptake dropped from roughly 1 in 3 for Asian women in those aged 46-49 years to only 1 in 6 aged ≥65 years.

After excluding those with previous breast cancer and not aged 46-73 years at entry, 879 Asian women and 51,779 white British/Irish women remained. Table 2 shows the age standardised risk factor summary at entry for women self-identified to be white British/Irish or Asian in the PROCAS study.

**Table 2:** Age standardised risk factor summary

Risk factor	Mean (standard deviation) or Percentage (%)^a^		Difference (95%CI)	P
	Asian	White	(Asian – White)	
Puberty				
Age at menarche (y)	13.3 (1.6)	12.9 (1.6)	0.37 (0.2 to 0.5)	< 0.001
Height and weight				
Body mass index (kg/m^2^)	26.5 (5.2)	27.5 (5.5)	−0.97 (−1.4 to − 0.6)	< 0.001
Weight (kg)	65.5 (13.4)	71.6 (14.8)	−6.10 (−7.1 to −5.1)	< 0.001
Height (m)	1.57 (0.07)	1.62 (0.07)	−0.045 (− 0.051 to − 0.039)	< 0.001
Reproductive factors				
Nulliparous	9.7%	13.0%	−3.39% (−5.5 to −1.0%)	0.003
Age first child(y)	24.7 (5.2)	24.4 (5.1)	0.36 (−0.08 to 0.8)	0.12
Children (n)	2.7 (1.7)	2.0 (1.2)	0.65 (0.5 to 0.8)	< 0.001
Four or more (y)	26.7%	10.0%	16.73% (13.1 to 20.4%)	< 0.001
1st <17y (y)	1.6%	1.6%	0.00% (−0.9 to 1.1%)	0.9
Menopausal status				
Pre-menopausal	14.5%	14.4%	0.15% (−1.6 to 1.9%)	0.9
Peri-menopausal	13.4%	16.6%	−3.17% (−5.2 to −1.1%)	0.002
Post-menopausal	72.0%	69.0%	3.01% (0.8 to 5.1%)	0.007
Age menopause (post-menopausal)	47.6 (5.7)	46.8 (5.9)	0.80 (0.3 to 1.3)	0.002
Hormone therapy (ever)	20.3%	38.3%	−18.02% (−21.1 to − 14.8%)	< 0.001
Genetic disposition				
1 or more affected first-degree relatives	10.4%	12.1%	−1.69% (−4.1 to 0.8%)	0.18
Mammographic density				
Percent density (%)	30.2 (18.1)	27.7 (17.3)	2.52 (1.1 to 4.0)	< 0.001
Percent density (%, BMI adjusted)	28.6^b^	26.8^b^	1.83 (0.04 to 3.3)	0.011
Overall 10-year risk (Tyrer-Cuzick model)				
Mean (standard deviation)	2.67 (1.39)	3.07 (1.49)	−0.405 (−0.53 to −0.26)	< 0.001
Percentage ≥ 5%	6.7%	9.9%	−3.28% (−5.3 to −1.1%)	0.003
Percentage < 2%	32.6%	18.9%	13.73% (10.2 to 17.3%)	< 0.001
Other risk factors				
Adult Weight gain (% change weight since 20y)	28.7 (22.2)	24.3 (21.3)	4.41 (3.4 to 6.0)	< 0.001
Physical Activity (y/n)	49.9%	72.2%	−22.29% (−26.7 to −18.0%)	< 0.001
Physical activity(> 4 h/wk)	58.0%	62.7%	−4.75% (−9.9 to 0.4%)	0.067
Drink alcohol (any)	18.6%	73.0%	−54.36% (−57.8 to − 50.8%)	< 0.001

^a^Mean (standard deviation) is used for continuous data, and percentage (%) for binary data

^b^Predicted (age-standardised) density for a woman with BMI 26-28 kg/m^2^

There were a number of differences in the incidence of risk factors that were reflected by a lower risk assessment for Asian women than white British/Irish women (mean 10 year risk 2.67% vs 3.07%). A greater proportion of Asian women fell into the low-risk (< 2% 10-year risk) group (32.6% vs 18.9%) and a lower proportion into the elevated risk (≥5% 10 year) group (6.7% vs 9.9%).

Asian women had a later age at menarche than white British/Irish women and were more likely to be post-menopausal with an earlier age at menopause. On average, Asian women were less obese (mean BMI 26.5 vs 27.5 kg/m^2^) and shorter (mean 1.57 vs 1.62 m) than white British/Irish women. Asian women were less likely to be nulliparous (9.7% vs 13.0%) and had larger families (26.7% had four or more children compared with 10.0%). In those with children, the age at first child was similar, as was the proportion with a first child aged < 17 years. Asian women were less likely to have a family history of breast cancer (10.4% vs 12.1% had one or more affected first-degree relatives), and much less likely to have ever used hormone therapy (20.3% vs 38.3%).

There were some differences in risk factors that are not included in the Tyrer-Cuzick risk assessment model. Alcohol use was substantially lower in Asian women than white British/Irish women (18.8% vs 73.0%). Asian women gained an absolute 4% more weight since age 20 years than white British/Irish women, and they were less likely to exercise (49.9% vs 72.2%). There was some evidence to suggest that mammographic density in Asian women was elevated compared to white British/Irish women, but the absolute difference was small after adjustment for age and BMI (1.8% greater, 95%CI 0.04 to 3.3%).

Cohort trends in risk factors that are expected to increase breast cancer incidence were seen in both the Asian and white British/Irish populations (Table 3).

**Table 3:** Risk factor trends by birth cohort and ethnic group

	Asian				White			
Cohort:	< 1950	1950-9	≥1960	P_trend_	< 1950	1950-9	≥1960	P_trend_
Number	157	375	347		18222*	21,111	12,424	
Age range (y)	60-73	49-64	46-55		60-73*	49-64	46-55	
Age at menarche (y)^a^	13.2 (1.6)	13.3 (1.7)	13.2 (1.6)	0.6	12.9 (1.6)	13.0 (1.5)	13.0 (1.7)	0.002
Body mass index^a^	25.8 (4.6)	27.6 (5.0)	27.3 (5.7)	0.29	27.3 (5.1)	27.2 (5.4)	27.4 (5.7)	< 0.001
Weight (kg)^a^	63.0 (11.5)	69.4 (14.1)	66.7 (13.9)	0.8	70.6 (13.6)	71.0 (14.7)	72.7 (15.5)	< 0.001
Height (m)^a^	1.56 (0.06)	1.59 (0.06)	1.56 (0.07)	0.047	1.61 (0.06)	1.61 (0.06)	1.63 (0.07)	< 0.001
Nulliparous^b^	6.3%	10.3%	13.1%	0.15	10.2%	15.4%	17.0%	< 0.001
≥4 children^b^	31.5%	25.9%	22.1%	0.004	12.3%	7.5%	8.1%	< 0.001
Age at first child (y)^a^	24.8 (5.2)	23.8 (4.9)	25.7 (5.1)	0.005	23.6 (4.3)	24.3 (5.2)	25.4 (5.6)	< 0.001
1st child <17y^b^	1.1%	2.3%	2.0%	0.18	0.8%	2.5%	2.3%	< 0.001
Number children (n)^a^	2.9 (1.6)	2.9 (2.0)	2.3 (1.7)	< 0.001	2.2 (1.3)	2.0 (1.2)	1.9 (1.3)	< 0.001
≥1 affected 1st-degree relative (%)^b^	15.1%	6.9%	6.4%	0.35	12.4%	9.7%	13.3%	0.11
Adult weight gain (%)^a^	27.8 (20.2)	31.1 (22.6)	33.3 (25.6)	0.9	24.7 (21.4)	24.2 (20.9)	22.8 (20.5)	< 0.001
Physical activity (y/n)^b^	52.0%	51.5%	39.9%	0.35	75.2%	73.8%	62.3%	< 0.001
Physical activity(> 17 h/month)^b^	60.2%	61.5%	43.7%	0.074	70.7%	58.5%	52.1%	< 0.001
Drink alcohol (y/n)^b^	20.4%	16.3%	14.2%	0.6	68.1%	77.9%	75.1%	< 0.001

*n = 41 white British/Irish women aged 58-59 years excluded from the table because not represented in Asian cohort born before 1950

^a^Mean (standard deviation) presented

^b^percentage presented

One of the largest changes was in the number of children and age at birth of first child. In the Asian group, there was a tendency for the older cohort to have given birth later and to have fewer children (31.1% in cohort born before 1950 had four or more children compared with 22.1% born ≥1960). There was also a doubling in nulliparity rates (6.3% to 13.1%). Similar trends were seen for white British/Irish women. Changes in frequency of physical activity were also observed; younger Asian and white British/Irish women were less likely to exercise. Alcohol use was most prevalent for white British/Irish women born after 1950. Due to the large sample size, many of the trends of other risk factors in white British/Irish women were statistically significant, but some differences were not large.

Rates of breast cancer by age group are presented in Table 4. Age standardised rates were lower in Asian women. Although this is in line with expectations from the analysis of risk factors, we lack statistical power to detect differences.

**Table 4:** Rates of breast cancer by age and ethnic group

	White				Asian				
	Women	Breast Cancer	Follow up^a^	Annual rate	Women	Breast Cancer	Follow up^a^	Annual rate	
Age (y)	N (%)	N (%)	(y)	(per 1000)	N (%)	N (%)	(y)	(per 1000)	Weights^b^
46-49	256 (29%)	2 (13%)	1042.4	1.9	10,380 (20%)	164 (15%)	39,679.8	4.1	22
50-54	289 (33%)	9 (60%)	1423.8	6.3	13,526 (26%)	246 (23%)	61,907.0	4.0	22
55-59	152 (17%)	2 (13%)	802.1	2.5	9735 (19%)	184 (17%)	48,530.3	3.8	20
60-64	112 (13%)	1 (7%)	579.8	1.7	9870 (19%)	277 (26%)	48,984.0	5.7	19
65-69	59 (7%)	1 (7%)	310.3	3.2	6700 (13%)	174 (16%)	33,371.6	5.2	17
70-73	11 (1%)	0 (0%)	54.1	0	1588 (3%)	31 (3%)	7819.4	4.0	0
Age-standardized (95% CI)				3.2 (1.6 - 5.2)				4.5 (4.2-4.8)	

^a^Follow up is the total number of women-years in each group from questionnaire to last follow up

^b^European standard population (2013) weights. Standardised rates only include women aged 46-69 years due to small number in Asian 70-73 years group

## Discussion

The present study has found that the predicted breast cancer risk attributable to some reproductive and hormonal risk factors is lower in Asian women than in the white British/Irish population attending mammographic screening in Greater Manchester. Asian women were more likely to be protected by multiple child births, absence of alcohol or hormone therapy use and a slightly later age at menarche. However, they were more likely to have increased risks related to larger adult weight gains and lower levels of physical activity.

The most thorough examination of ethnicity and breast cancer risk in the UK to date was analysis based on the Million Women Study (MWS). This recruited women, on average, 13 years earlier than in the PROCAS study [16]. It found that South Asian women had a reduced breast cancer incidence compared with white women (relative risk 0.82, 95% CI 0.72–0.94). By way of comparison, in this study the average 10 year standardised predicted relative risk for Asian vs white women was 2.67/3.07 = 0.87.

PROCAS is a more recent birth cohort than in the MWS, in which the youngest women were born in 1951. Thus the PROCAS and MWS cohorts represent different generations of Asian women. Furthermore, it is possible that the majority of Asian women in the MWS were born and brought up in Asia, as mass migration to the UK only began after 1947. We do not have data on place of birth in PROCAS, but it is probable that most Asian women in PROCAS were born or resided in the UK during pubertal development. Approximately 5.3% of the Asian group in the MWS reported a first-degree relative with breast cancer, compared with 9.5% of the white group. In the present study, the age-standardised percentage was 10.4% of Asian women compared with 12.1% of the white British/Irish population. The much closer percentages between ethnic groups in our study might reflect that previous generations in Asia had much lower breast cancer incidences and shorter lifespans. We found a small increase in mammographic density in Asian women compared to white British/Irish women after adjustment for age and BMI, which is in line with previous studies [17].

Some important trends in risk factors for Asian and white British/Irish women were observed, for instance, later ages at first birth were observed within the more recent birth cohorts. Previous research has indicated that part of the change in incidence in Asian populations who migrate to Western countries might be due to changes in parity [16]. The trend in the Asian group here was quite clear, with a doubling of nulliparity rates, fewer children and a later age at first birth. In addition, adult weight gain is becoming an established predictor of post-menopausal breast cancer risk [18]. Asian women had proportionally greater adult weight gains than white British/Irish women and they were also far less likely to undertake regular physical activity or meet physical activity guidelines, particularly in the more recent birth cohort. This pattern is also expected to lead to increasing breast cancer risk for Asian women, including those women who are currently pre-menopausal.

The current study has several limitations. There was some missing data in the questionnaire (see Additional file 1), and we only used fully complete fields. Methods to assess physical activity and weight change were rather crude, and more refined follow-up studies could be planned. Recruitment to PROCAS depended on women attending breast screening and volunteering to join the study. Women who do not attend screening may have different risk factor profiles than those who do. For example, attendance is linked with socio-economic status which in turn is also linked with increased rates of overweight and obesity. Uptake to PROCAS was higher in more affluent areas. However, the issue of non-attendance affects both ethnic groups examined here, so the analysis is best interpreted as risk factors in women who attend screening. Further, uptake was similar to that in the MWS. But overall, an important limitation is it is possible that Asian women who joined PROCAS are not representative of the Asian population in Greater Manchester. Asian women only represented 1.5% of the total PROCAS population and the numbers of breast cancers in this group were small. Nonetheless, they probably represent a population of younger Asian women who attend screening and have been born or brought up in the UK. It is possible that differences in risk factor distributions are not very generalizable beyond the study, and that differences may also arise due to a relatively small sample of Asian women. However, many of the differences are large and consistent with prior expectations (e.g. Asian women were shorter, less obese and less likely to drink alcohol), and some of the differences are quite large, so there is some qualitative support that the differences may well be generalizable.

Breast cancer is largely attributable to non-genetic factors. For example, studies have shown that women born in areas with low rates of the disease have low rates of the disease when they migrate to a country with higher rates, but that the risk in subsequent generations increases towards the rate of women born in the country [5]. We observed a lower predicted risk of breast cancer in women with an Asian heritage in this study. Unfortunately the study lacked statistical power to test whether this risk assessment is accurate. Additional follow-up of the PROCAS cohort and further studies are warranted to help assess these aspects.

## Conclusion

In conclusion, the current study suggests that Asian women remain at lower risk of breast cancer than white British/Irish women. However, trends in risk factors in both populations suggest an ongoing increase in breast cancer risks. For Asian women, preventive interventions might focus on reducing risks from modifiable risk factors such as weight reduction and increasing levels of physical activity.

## Additional file

Additional file 1: PROCAS questionnaire. (DOCX 17 kb)

## Abbreviations

BMI: Body mass index
GMBSP: Greater Manchester Breast Screening Programme
MWS: Million women study
NHS: National Health Service
NHSBSP: National Health Service Breast Screening Programme
NIHR: National Institute for Health Research
PROCAS: Predicting Risk of Cancer at Screening
UK: United Kingdom
VAS: Visual analogue scale
