# Supplementary material for: Breast cancer risk in a screening cohort of Asian and white British/Irish women from Manchester UK
Source: BMC Public Health. 2018 Jan 25;18:178. doi: 10.1186/s12889-018-5090-9 (PMC5784727; doi:10.1186/s12889-018-5090-9)
Supplement: Additional file 1: — PROCAS questionnaire. (DOCX 17 kb) [file 12889_2018_5090_MOESM1_ESM.docx]

**Supplementary table**

The number of unknown fields for each risk factor and cohort are shown in the table below.

**Unknown fields by ethnic group and cohort (companion to Tables 2/3).**

|  | **British Asian** | | | |  | **British White** | | | |
| --- | --- | --- | --- | --- | --- | --- | --- | --- | --- |
|  | **Overall** | **<1950** | **1950-59** | **1960** |  | **Overall** | **<1950** | **1950-59** | **1960** |
| **Total number** | 879 | 157 | 375 | 347 |  | 51779* | 18222** | 21111 | 12424 |
| **Menarche (y)** | 50 (5.7%) | 12 (7.6%) | 22 (5.9%) | 16 (4.6%) |  | 1062 (2.1%) | 365 (2.0%) | 437 (2.1%) | 260 (2.1%) |
| **BMI (kg/m2)** | 107 (12.2%) | 17 (10.8%) | 44 (11.7%) | 46 (13.3%) |  | 3295 (6.4%) | 941 (5.2%) | 1295 (6.1%) | 1056 (8.5%) |
| **Weight (kg)** | 91 (10.4%) | 14 (8.9%) | 38 (10.1%) | 39 (11.2%) |  | 2783 (5.4%) | 725 (4.0%) | 1128 (5.3%) | 929 (7.5%) |
| **Height (m)** | 44 (5.01%) | 9 (5.73%) | 22 (5.87%) | 13 (3.75%) |  | 889 (1.72%) | 318 (1.75%) | 304 (1.44%) | 265 (2.13%) |
| **Nulliparous** | 0 (0.0%) | 0 (0.0%) | 0 (0.0%) | 0 (0.0%) |  | 6 (<0.1%%) | 0 (0.0%) | 1 (<0.1%%) | 5 (<0.1%%) |
| **4+ children** | 0 (0.0%) | 0 (0.0%) | 0 (0.0%) | 0 (0.0%) |  | 6 (<0.1%%) | 0 (0.0%) | 1 (<0.1%%) | 5 (<0.1%%) |
| **Age first(y)** | 11/788 (1.4%) | 4/148 (2.7%) | 5/331 (1.5%) | 2/309 (0.6%) |  | 92/44835 (0.2%) | 29/16366 (0.2%) | 44/18069 (0.2%) | 19/10364 (0.2%) |
| **1st <17y** | 102 (11.6%) | 13 (8.3%) | 49 (13.1%) | 40 (11.5%) |  | 7050 (13.6%) | 1885 (10.3%) | 3085 (14.6%) | 2074 (16.7%) |
| **Children (n)** | 0 (0.0%) | 0 (0.0%) | 0 (0.0%) | 0 (0.0%) |  | 22 (<0.1%%) | 5 (<0.1%%) | 10 (<0.1%%) | 7 (<0.1%%) |
| **Affected rel (1+ 1st-deg %)** | 0 (0.0%) | 0 (0.0%) | 0 (0.0%) | 0 (0.0%) |  | 0 (NA%) | 0 (0.0%) | 0 (0.0%) | 0 (0.0%) |
| **Weight gain (%)** | 260 (29.6%) | 52 (33.1%) | 106 (28.3%) | 102 (29.4%) |  | 6582 (12.7%) | 1950 (10.7%) | 2582 (12.2%) | 2047 (16.5%) |
| **Physical Activity (y/n)** | 0 (0.0%) | 0 (0.0%) | 0 (0.0%) | 0 (0.0%) |  | 2 (<0.1%%) | 1 (<0.1%%) | 1 (<0.1%%) | 0 (0.0%) |
| **Physical activity(>17h/mo)** | 50 (5.7%) | 86 (54.8%) | 178 (47.5%) | 203 (58.5%) |  | 2203 (4.3%) | 4829 (26.5%) | 6162 (29.2%) | 4758 (38.3%) |
| **Drink alcohol** | 9 (1.0%) | 0 (0.0%) | 7 (1.9%) | 2 (0.6%) |  | 583 (1.1%) | 236 (1.3%) | 201 (1.0%) | 146 (1.2%) |
| **Pre-meno** | 104 (11.8%) |  |  |  |  | 2684 (5.2%) |  |  |  |
| **Peri-meno** | 104 (11.8%) |  |  |  |  | 2684 (5.2%) |  |  |  |
| **Post-meno** | 104 (11.8%) |  |  |  |  | 2684 (5.2%) |  |  |  |
| **Age meno** | 99/469 (21.1%) |  |  |  |  | 4925/33232 (14.8%) |  |  |  |
| **HRT** | 21 (2.4%) |  |  |  |  | 435 (0.8%) |  |  |  |
| **TC (10y%)** | 0 (0.00%) |  |  |  |  | 1 (<0.1%%) |  |  |  |
| **TC (5%+ 10y)** | 0 (0.0%) |  |  |  |  | 1 (<0.1%%) |  |  |  |
| **TC (<2% 10y)** | 0 (0.0%) |  |  |  |  | 1 (<0.1%%) |  |  |  |
